# Malaria in a metropolitan region of Southern Germany: past, present and future perspectives on a protozoan infection with the potential of re-appearance in Central Europe

**DOI:** 10.1186/s12936-025-05292-y

**Published:** 2025-02-22

**Authors:** Jan Esse, Karl-Heinz Leven, Helge Kampen, Doreen Werner, Silke Göttler, Christian Bogdan

**Affiliations:** 1 Mikrobiologisches Institut - Klinische Mikrobiologie, Immunologie und Hygiene, Universitätsklinikum Erlangen and Friedrich-Alexander-Universität (FAU) Erlangen-Nürnberg, Wasserturmstraße 3/5, 91054 Erlangen, Germany; 2 Institute for the History and Ethics of Medicine, FAU Erlangen-Nürnberg, Glückstraße 10, 91054 Erlangen, Germany; 3 Friedrich-Loeffler-Institut, Federal Research Institute for Animal Health, Südufer 10, 17493 Greifswald - Insel Riems, Germany; 4 Leibniz Centre for Agricultural Landscape Research, Eberswalder Straße 84, 15374 Muencheberg, Germany; 5 Biogents AG, An Der Irler Höhe 3a, 93055 Regensburg, Germany; 6 FAU Profile Center Immunomedicine, FAU Erlangen-Nürnberg, Freyeslebenstraße 1, 91054 Erlangen, Germany

**Keywords:** Autochthonous transmission, *Plasmodium vivax*, *Anopheles maculipennis*, *Anopheles plumbeus*, *Anopheles daciae*, *Anopheles claviger*, *Anopheles messeae*, Bavaria, Globalization, Climate change

## Abstract

**Background:**

Malaria occurred endemically in Germany until the twentieth century. Climate change and globalization are known to promote the spreading of malaria. Erlangen is a city with just under 120,000 inhabitants located in the Nürnberg metropolitan region, Federal State of Bavaria, Southern Germany. Historical findings, current climate data, microbiological data (local and state level) and vector surveillance data are used to estimate the risk of re-emergence and autochthonous transmission of malaria in the area of Erlangen.

**Methods:**

Historical data was obtained by searching literature. Climatic data were retrieved from the German Climate Data Centre. Data on reported (supra-)regional infections were obtained from the Robert-Koch Institute. Cases of malaria diagnosed at the Institute of Clinical Microbiology, Immunology and Hygiene (University Hospital Erlangen) complement this data. The citizen science project “Mückenatlas” (Mosquito Atlas), the German mosquito database (CULBASE) and the company Biogents AG provided mosquito surveillance data.

**Results:**

Malaria was highly endemic in Erlangen in the nineteenth century, with 18% of hospitalized patients suffering from this disease in 1860, but disappeared during the first half of the twentieth century. After the end of World War II, autochthonous ‘malaria tertiana’ (tertian malaria) occurred in neighbouring Nürnberg, demonstrating the regional malaria potential. In recent decades, the average monthly temperature increased by 1.6 °C. In Erlangen and the surrounding area, three potential vectors of tertian malaria parasites are prevalent (*Anopheles messeae, Anopheles maculipennis *sensu stricto, and *Anopheles plumbeus*). In addition, *Anopheles daciae,* which has unknown potential of *Plasmodium* transmission, and *Anopheles claviger *sensu lato have been detected. In recent years, malaria diagnosed in Erlangen mainly resulted from travelling to Africa. *Plasmodium vivax* accounted for only a small proportion of these cases (2010–2023: n = 5, 17%).

**Conclusion:**

Future autochthonous transmission of malaria parasites in Erlangen is possible, although re-establishment of a natural transmission cycle is currently unlikely. In order to avoid unexpected autochthonous malaria, surveillance and prevention measures should be considered. Patients with fever after visiting endemic areas need to be analysed for *Plasmodium* infection.

## Background

Malaria is one of the worldwide most important infectious diseases. In 2022, it was endemic in 85 countries and caused around 249 million infections and 608,000 deaths. The majority of infections (94%) originates from Africa [1]. Malaria is caused by protozoa of the genus *Plasmodium*, which are transmitted by mosquitoes of the genus *Anopheles*. Transmission is also possible by medical procedures [2–4] and by intravenous drug abuse [5]. Human pathogenic parasite species are *Plasmodium falciparum*, *Plasmodium vivax*, *Plasmodium ovale*, and *Plasmodium malariae,* but humans can also be infected by some primate malaria parasites, including *Plasmodium knowlesi* [6]. Remarkably, the share of *P. vivax*, the prevailing cause of ‘malaria tertiana’ (tertian malaria), amongst all malaria infections has diminished significantly since 2000 (2000: 20.5 million, 8%; 2022: 6.9 million, 3%) [1]. Tertian malaria is characterized by a fever cycle of 48 h and is usually benign. However, both *P. vivax* and *P. ovale* infections*,* although self-limiting, may cause later relapses due to the persistence of hypnozoites in hepatocytes, if not treated properly [6].

### Malaria in Germany and other non-endemic countries

For a long time, malaria, especially tertian malaria, was highly endemic in Europe [7–9] and in Germany [10–12]. Around 1850, malaria incidence rose in many places in Germany, but afterwards steadily decreased until the end of the nineteenth century [13]. Causative factors for the decline and disappearance of autochthonous malaria include the consistent treatment of parasite carriers with quinine [11], the drainage of wetlands [12] as well as changing living conditions, in particular the separation of living rooms and stables [13–15]. Increasing host immunocompetence due to improved nutrition of the population [15] or genetic alterations leading to reduced virulence of the parasite [10] could also have contributed to the decline of malaria in Germany. Although local and temporary outbreaks of malaria were reported in Germany after World War I and II [15, 16], a long-term re-establishment of malaria was not observed. Since tertian malaria did not only occur in Germany, but also in Scandinavia and Russian Karelia during the nineteenth and twentieth century [17, 18], it is historically not a tropical disease. It can also be assumed that the typical clinical presentation of tertian fever was well known to medical doctors in Germany.

Although malaria is currently no longer endemic in Germany [19], *Plasmodium* parasites can be imported in various ways. Non-autochthonous cases of malaria in non-endemic countries are mostly due to infected travelers or immigrants [20]; rarely, they result from the passive transfer of *Anopheles* infected with *Plasmodium* species (spp.) via aircrafts (airport malaria) or baggage [21, 22]. If an index case becomes the starting point for transmission of *Plasmodium* spp. by a local vector to a second person, criteria of autochthonous malaria are met. Since eradication, several clusters of autochthonous tertian malaria have occurred both in Europe [23–26] and in the USA [27] without any long-term epidemic following to date. However, infected *Anopheles* populations have been found in close proximity to malaria cases in the USA [28].

### Climate change

As *Anopheles* spp. are ectothermic organisms, their evolution and prevalence as well as the development of ingested *Plasmodium* spp. are driven by the ambient temperature [29]. The average monthly temperature needed for the extrinsic development of *P. vivax* is around 16 °C (range 14.5–17.5 °C) [29–31]. The optimum temperature for both sporozoite maturation and *Anopheles* development, which initially accelerates with rising temperature, is between 24 and 30 °C, whereas above 30 °C the survival and development of *Anopheles* is restricted [31]. Thus, increased rates of resurgence and spread of tertian malaria can be expected in the context of global warming [32].

## Methods

### Setting

Erlangen is a city in the German Federal State of Bavaria (population size as of December 31, 2023 was 117,806) [33], which belongs to the Nürnberg metropolitan region within the administrative district of Middle Franconia (49° 35′ 32" N, 11° 0′ 34" E) [34].

### Literature search

For this work, mainly primary source and research literature was consulted. Some documents were examined from the holdings of the Erlangen City Archives, but no material relating to malaria was found (archive records examined: 6.A.121, 6.A.122, F1247/10, F147/11, F147/12, F147/13, 9.A.192 and 9.A.1214). In addition, all issues of the daily newspaper “Erlanger Tagblatt” from the year 1899 were assessed [35]. The “Erlanger Tagblatt” contained monthly overviews of infectious diseases reported by the Erlangen district office and the city of Erlangen at irregular intervals. These overviews also listed the term “Wechselfieber” (intermittent fever), which was used synonymously for malaria.

### Erlangen malaria districts

Mayr divided the city of Erlangen 1889 into several districts with regard to the historical occurrence of malaria based on the elevation and the location within the city: I. District: one metre and more above the level of the train station; II. District: even to or up to one metre above the level of the train station; III. District: even to or up to one metre below the level of the train station; IV. District: one to five metres below the level of the train station; V. District: variable elevation, city district “Essenbach”; VI. District: up to 20 m below the level of the train station, city district “Werke” [36]. The given elevations were compared to current data (Google Earth V 10.43.0.2, 2023. Erlangen, Germany. https://earth.google.com/web/, accessed 2023.12.15) and transferred to a historical map of the year 1890 [37]. The map was digitized by the Bavarian State Library (https://mdz-nbn-resolving.de/urn:nbn:de:bvb:12-bsb00105102-0, accessed 2023.12.15) and edited within the scope of the terms of use (CC BY-NC-SA 4.0 DEED, https://creativecommons.org/licenses/by-nc-sa/4.0/, accessed 2023.12.15).

### Climate data

Climate data used correspond to the monthly average air temperature (°C) at an altitude of 2 m (station 1279—Möhrendorf-Kleinseebach, data set ID: urn:x-wmo:md:de.dwd.cdc:: OBS_DEU_P1M_T2M). The data was retrieved from the online portal of the German Climate Data Centre (Deutscher Wetterdienst, Offenbach, Deutschland, CDC-v2.1.b22.09) [38].

### Mosquito surveillance

In order to obtain an overview of the non-native mosquito species occurring in Bavaria, in particular the “Asian tiger mosquito” (*Aedes albopictus*), the Bavarian Mosquito Monitoring Scheme was initiated in 2022 by the Bavarian State Ministry of Health and the Bavarian State Ministry of the Environment and Consumer Protection (carried out by the Bavarian Health and Food Safety Authority [LGL]). The LGL is not aware of *Anopheles* findings in the city of Erlangen, as Erlangen was not specifically monitored and no submissions were received from the population of Erlangen up to now (March 18, 2024, personal communication, S. Böhm, LGL). In addition to the Bavarian investigations, a nationwide citizen science project, the so-called “Mückenatlas” (Mosquito Atlas), was initiated in the year 2012 (https://mueckenatlas.com/, accessed 2024.10.02) [39]. Citizen submissions, trap collections and targeted sampling studies feed the German mosquito database (CULBASE) at Leibniz Centre for Agricultural Landscape Research (ZALF, Muencheberg, Germany). The mosquitoes listed in the CULBASE database were identified morphologically at ZALF and molecularly at the Friedrich-Loeffler-Institute (Greifswald—Insel Riems, Germany). A database query for the years 2011 to 2023 was carried out targeting vector-competent *Anopheles* spp. For this purpose, a radius of 2 km around the area of the Erlangen city district was defined. The municipalities concerned were included in the evaluation (the furthest extent of the included municipal areas results in a minimum distance of the outer boundary to the border of the Erlangen city district of around 4 km). The selected study area was determined by the assumed flight range of the vectors expected in this region (e.g., *Anopheles maculipennis* complex) [40, 41]. In addition, data from the mosquito surveillance commissioned by the neighbouring city of Fürth, carried out by Biogents AG (Regensburg, Germany), was analysed for the years 2021 to 2023. These data were obtained as part of a standardized investigation in the “Südstadt” district (zip code 90763; distance of the nearest traps to the border of Erlangen city district approx. 8.2 km). For this purpose, attractant traps (BG-Pro, Biogents AG) equipped with a CO_2_ source were set up from April to November, approximately every 2 weeks for 24 h each. The captured mosquitoes were morphologically differentiated on species level.

**Fig. 1:**
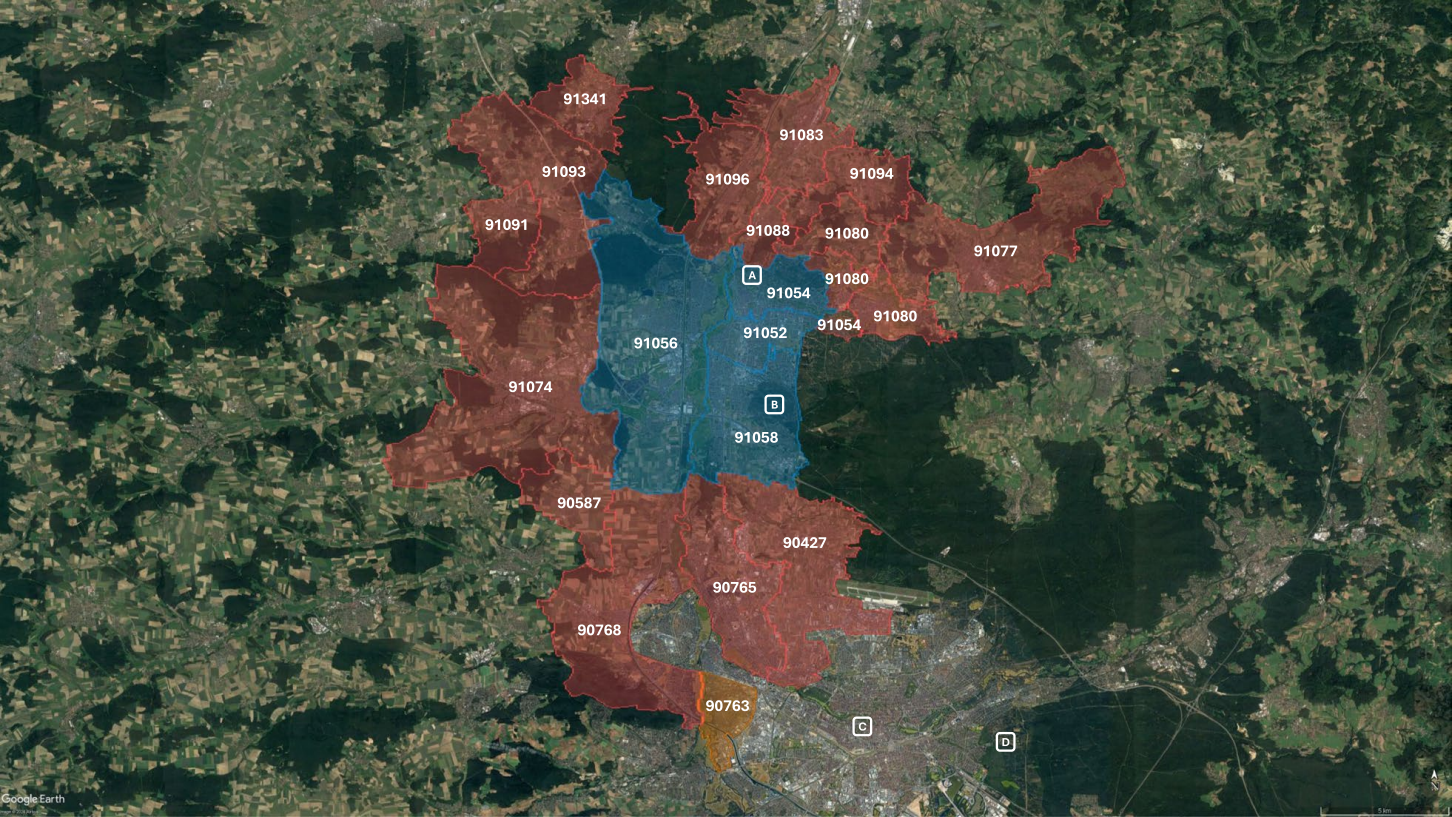
Zip code areas assessed regarding the occurrence of *Anopheles* species. Blue, city districts of Erlangen; red, zip code areas less than 2 km away from the city districts of Erlangen; orange, Fürth Südstadt; ^a^ Eisgrube; ^b^ Brucker Lache; ^c^ Nürnberg Imperial Castle; ^d^ Nürnberg Zoo. For the results of the assessment, see Table 4 and Fig. 6. Map data: ©2024: Google, Airbus, GeoBasis-DE/BKG [©2009] and ©OpenStreetMap contributors, https://www.openstreetmap.org/copyright/en, retrieved Febr. 20, 2024

The zip code areas (Fig. 1; KML files retrieved on Feb 16, 2024, from https://www.suche-postleitzahl.org/, data as of July 15, 2023) are based on freely available datasets (©OpenStreetMap contributors, https://www.openstreetmap.org/copyright/en, retrieved Feb. 20, 2024). The files were imported into Google My Maps and then displayed in Google Earth Pro (version 7.3.6.9750; map data ©2024: Google, GeoBasis-DE/BKG [©2009], Airbus).

### Government surveillance data

Until July 2023, the diagnosis of malaria in Germany was reported without mentioning the patients’ names to the Robert-Koch Institute (RKI), the central federal institution for disease prevention and control in Germany, in accordance with §7.3 of the Infection Protection Act (Infektionsschutzgesetz, IfSG). Since then, the responsible health authority had to be notified about cases of malaria along with the patients’ names in accordance with §7.1 IfSG. The nameless notifications (from 2001 to 7/2023) and the named reports (since 8/2023) on malaria patients with main residence in Bavaria were retrieved using the RKI online tool SurvStat@RKI 2.0 (SurvStat@RKI 2.0, https://survstat.rki.de, query date: 2024.03.18). An RKI overview of the malaria cases reported nationwide in 2022 allowed a comparison with microbiological and anamnestic data of *Plasmodium* infections diagnosed in Erlangen [42].

### Microbiological data

The results of all parasitological tests for malaria carried out at the Institute for Clinical Microbiology, Immunology and Hygiene of the University Hospital Erlangen, a maximum-care hospital with over 1,400 beds, were obtained for the years 2010 to 2023 using the Microsoft^®^ Excel^®^-based statistics module of the laboratory information software SWISSLAB (NEXUS SWISSLAB GmbH, Berlin, Germany, Version 2.22.7.000016). Each patient was counted only once. The parasitological diagnosis of malaria was based on Giemsa-stained thin and thick peripheral blood films and on immunochromatographic tests.

### Statistical analysis

Statistical analyses were performed as a two-tailed Fisher exact test using SPSS, V29.0.1.0 (IBM, Armonk, USA). Average values were calculated using Microsoft^®^ Excel^®^ 2016 MSO (16.0.5422.1000, Microsoft Corporation, Redmond, USA).

## Results

### Malaria in Erlangen—historical perspective

**Fig. 2:**
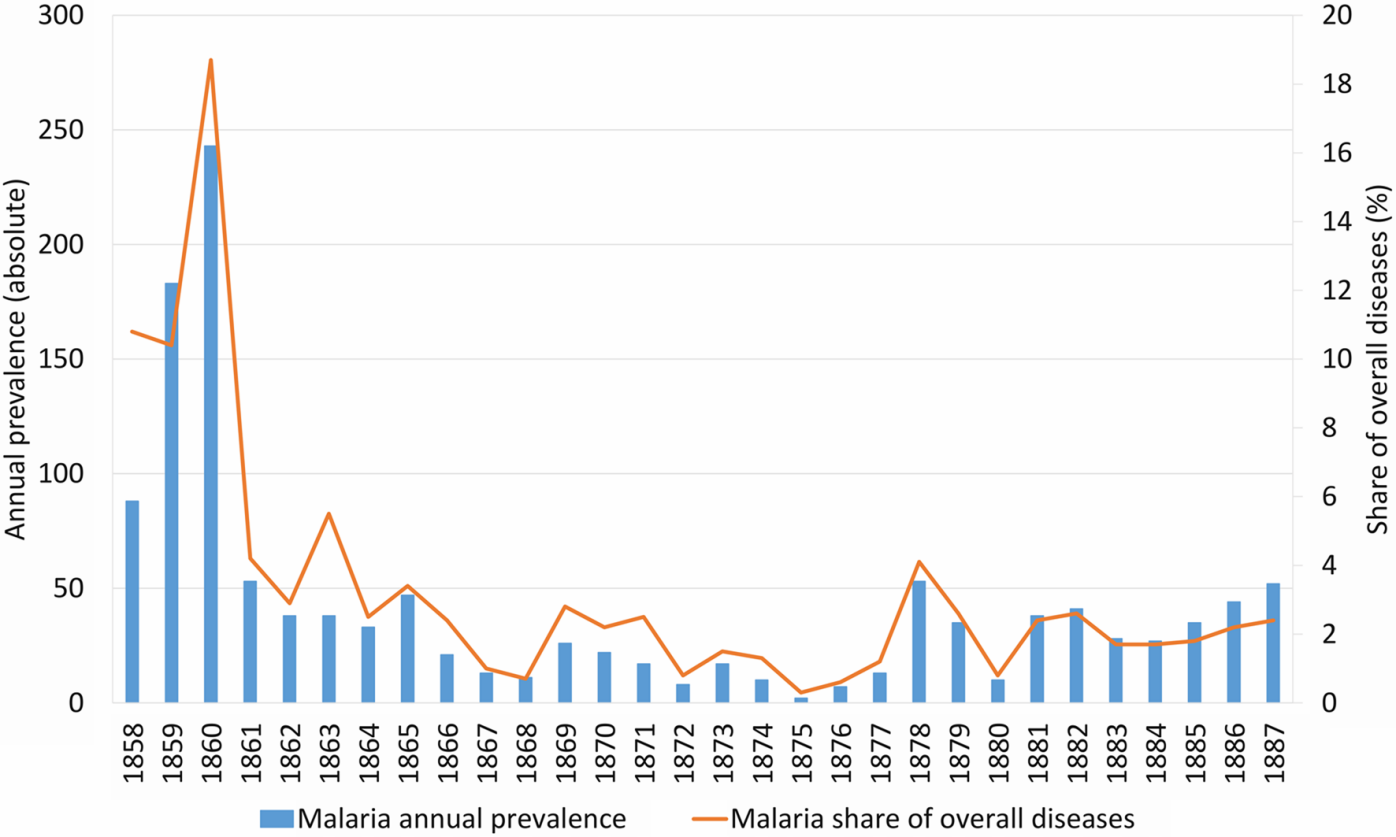
Malaria case numbers in Erlangen, 1858 to 1887. Data according to Mayr [36]

The majority of the malaria cases for the city of Erlangen were reported 1889 by Mayr in his dissertation “Malaria in Erlangen over the past 30 years” [36]. Data are partly based on earlier studies by Penzoldt from 1883 [43]. From 1858 to 1860, the incidence of malaria increased with an annual maximum registered in 1860 (n = 243, 18.7% of hospitalized patients). Although malaria cases were observed every year thereafter, they accounted for a significantly lower proportion of hospitalized patients (Table 1, Fig. 2).

**Table 1:** Malaria case numbers in Erlangen, 1858–1903*

Year	Patients diagnosed with malaria	Share of diagnoses (%)
1858	88	10.8
1859	183	10.4
1860	243	18.7
1861	53	4.2
1862	38	2.9
1863	38	5.5
1864	33	2.5
1865	47	3.4
1866	21	2.4
1867	13	1.0
1868	11	0.7
1869	26	2.8
1870	22	2.2
1871	17	2.5
1872	8	0.8
1873	17	1.5
1874	10	1.3
1875	2	0.3
1876	7	0.6
1877	13	1.2
1878	53	4.1
1879	35	2.6
1880	10	0.8
1881	38	2.4
1882	41	2.6
1883	28	1.7
1884	27	1.7
1885	35	1.8
1886	44	2.2
1887	52	2.4
n/d	n/d	n/d
1898	7	n/a
1899	18	1.1
1900	2	n/a
n/d	n/d	n/d
1903	6	n/a

^*^1858 to 1887: malaria cases treated as in-patients according to Mayr 1889 [36]; 1898, 1900 and 1903: without further specification according to Schuberg 1927 [44]; 1899: cases treated as out-patients according to information in the Erlanger Tagblatt [35]; n/d, no data available; n/a, not applicable as reference values are missing

**Fig. 3:**
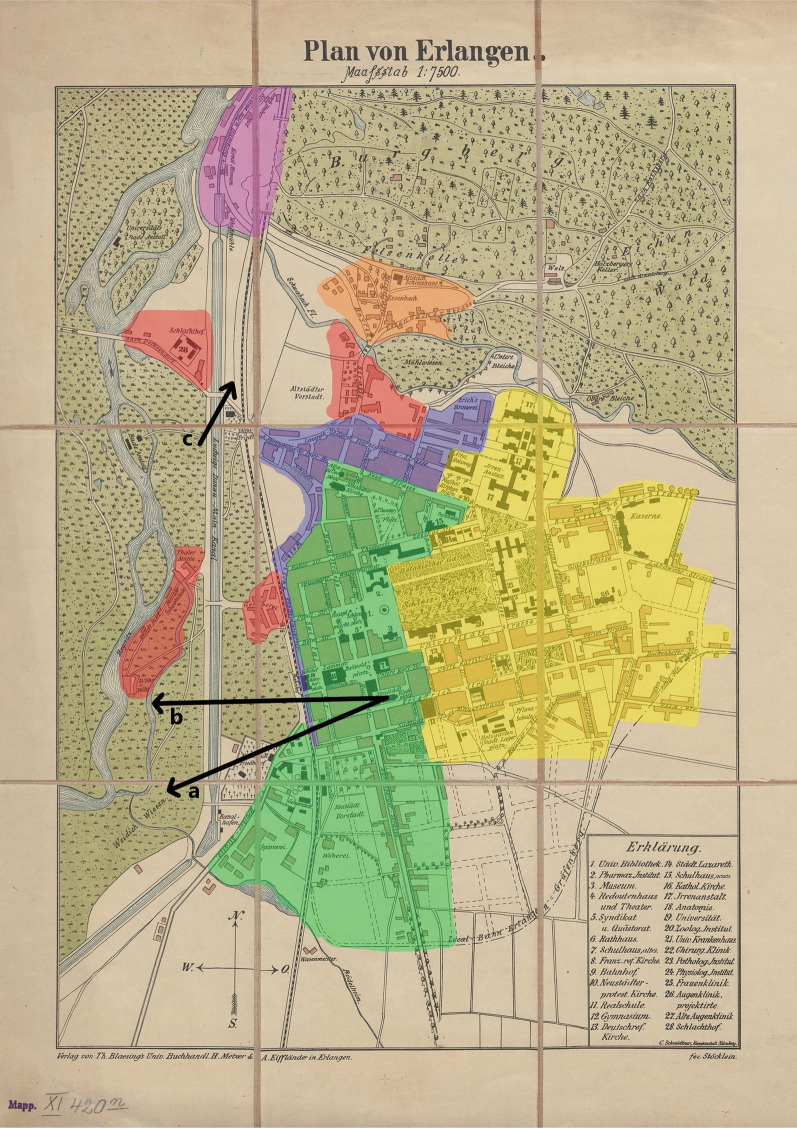
Erlangen districts categorized according to malaria case numbers. Green, “District I”; yellow, “District II”; blue, “District III”; red, “District IV”; orange, “District V”; violet, “District VI”. Classification of the districts according to Mayr 1889 (see Methods) [36]. The number of cases per district calculated by Penzoldt 1883 shows an accumulation of malaria cases in districts V and VI (see Table 2) [43]. ^a^Approximate line of sight of Fig. 7; ^b^approximate line of sight of Fig. 8; ^c^approximate line of sight of Fig. 9. Based on Stöcklein H. [Map of Erlangen], Erlangen; Nürnberg: Blaesing; Schmidtner; 1890

Of the six malaria districts of Erlangen, two were particularly affected (Table 2). These were the northern districts “Essenbach” (district V) and the so-called “Werke” (district VI), which were significantly below the altitude of the rest of the city (see Fig. 3 for an illustration of the districts). After 1887, only sporadic cases of malaria were reported in individual, disconnected years [44]. In 1899, the “Erlanger Tagblatt” reported malaria cases for the district of Erlangen comparable to the numbers in the 1880s reported by Mayr (Table 1). In neighbouring Nürnberg, the dynamic of malaria cases in the 19th and early twentieth century was comparable to the situation in Erlangen (Table 3). After the Second World War, however, especially in 1946 and 1947, a number of autochthonous tertian malaria cases occurred in Nürnberg, which were analysed aetiologically in a dissertation from 1949. An autochthonous nature of the infections were very likely, as the patients had not stayed in classical risk areas [45].

**Table 2:** Malaria case numbers in various districts of the city of Erlangen, 1866–1883*

District	Overall disease cases	Inpatient cases diagnosed with malaria	Share of inpatient diagnoses (%)
I	5,658	61	1.0
II	7,500	108	1.4
III	3,576	58	1.6
IV	3,600	63	1.8
V	966	41	4.2
VI	318	41	12.9
Total	21,618	372	1.7

^*^Adapted from Penzoldt 1883, who described the cases of malaria as “intermittent illnesses” (“Intermittenserkrankungen”) [43]. The total number of cases was recorded for three years and then extrapolated to the 18-year period from 1866 to 1883. For a detailed description of the given districts, see Methods section and Mayr (1889) [36]. The geographical distribution of the districts is shown in Fig. 3

**Table 3:** Malaria case numbers in Nürnberg, 1849–1953*

Year	Patients diagnosed with malaria	Year	Patients diagnosed with malaria
1849	10^a^	1890	195
1850	35^a^	1891	165
1851	30^a^	1892	109
1852	19^a^	1893	124
1853	48^a^	1894	82
1854	61^a^	1895	69
n/d	n/d	1896	60
1859	60^b^	1897	38
n/d	n/d	1898	40
1862	39^b^	1899	29
1863	24^b^	n/d	n/d
1864	24^b^	1909	8
1865	11^b^	n/d	n/d
1866	20^b^	1912	4
1867	39^b^	n/d	n/d
1868	29^b^	1920	2
1869	62^b^	1921	5
1870	52^b^	n/d	n/d
1871	62^b^	1945	12^d^
1872	28^b^	1946	31^c^ or 23^d^
1873	38^b^	1947	16^c^ or 15^d^
1874	18^b^	1948	23
n/d	n/d	1949	6
1881	164^b^	1950	2
1882	229^b^	1951	1
1883	218	1952	1
1884	216	1953	1
1885	169		
1886	127		
1887	135		
1888	129		
1889	156		

^*^^a^Patients treated at the local clinic; ^b^ patients treated at the municipal hospital; the remaining data refer to the city of Nürnberg in general, without mentioning a specific place of treatment; n/d, no data available; 1849 to 1921 according to Schuberg 1927 [44]; ^c^ 1946 to 1947 according to Steib 1949 [45]; ^d^ 1945 to 1947 according to Hormann 1949 [78]; 1948 to 1953 according to Heber 1990[79]

### Malaria in Erlangen—present situation

**Fig. 4:**
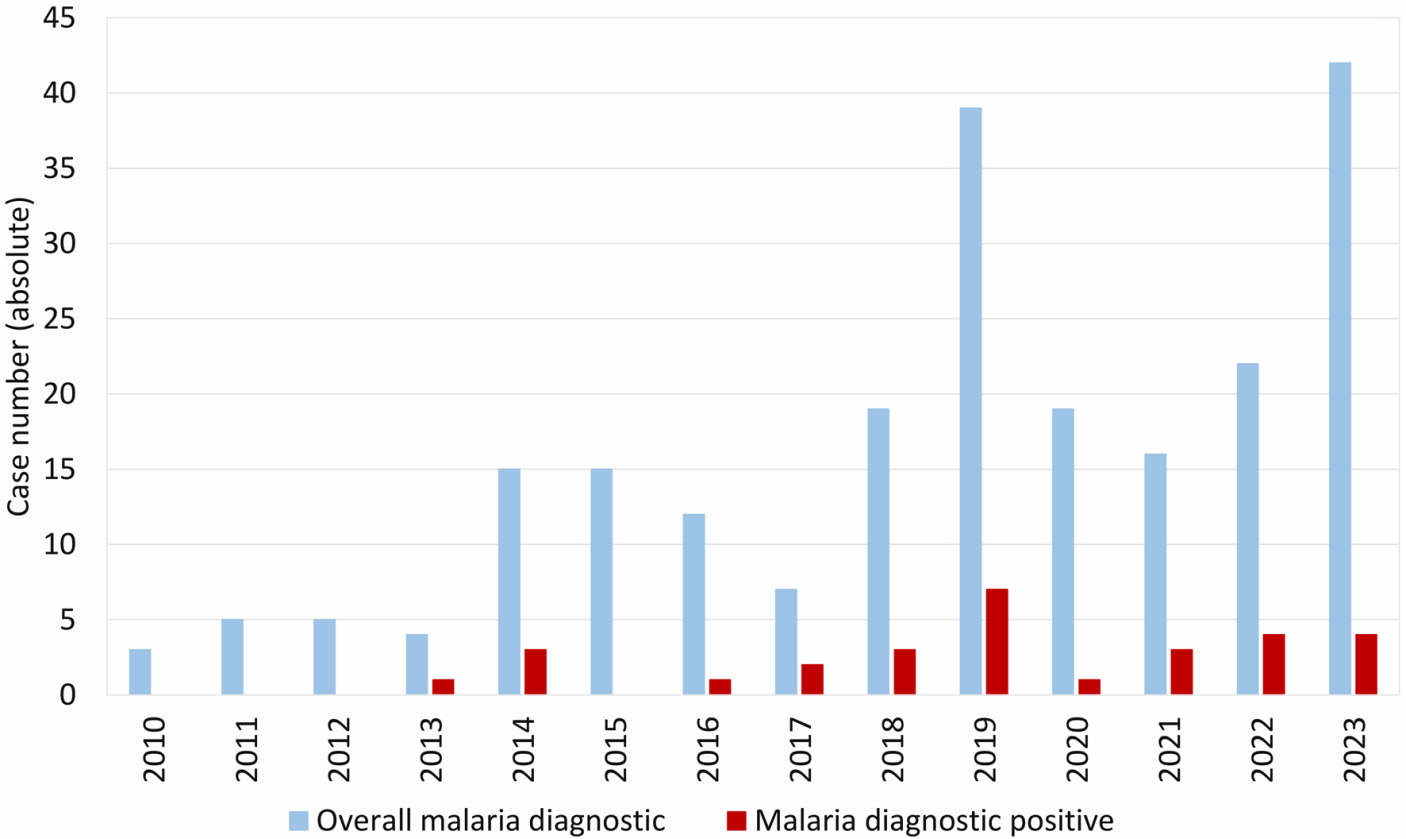
Diagnoses of malaria at the University Hospital Erlangen, 2010–2023. Total number of patient samples tested for malaria and proportion of detection of *Plasmodium* parasites. Each patient was only counted once

**Fig. 5:**
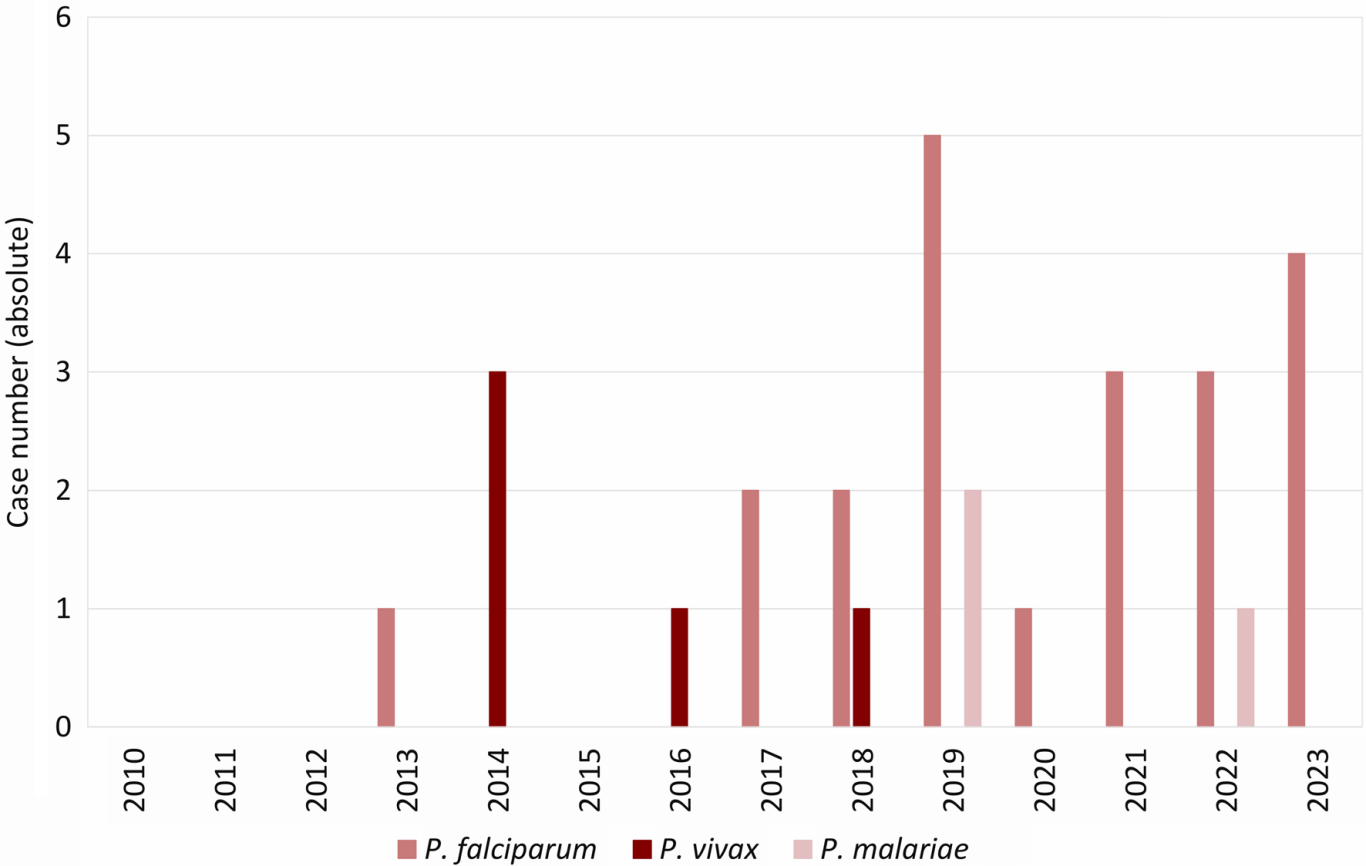
*Plasmodium* species detected at the University Hospital Erlangen, 2010–2023. Each patient was counted only once. *P.*, *Plasmodium*. No other than the given *Plasmodium* species was detected in the assessed period

At the Institute for Clinical Microbiology, Immunology and Hygiene of the University Hospital Erlangen, a total of 223 peripheral blood tests for suspected malaria were carried out from 2010 to 2023 with an increasing trend (2010 n = 3, 2023 n = 42; Fig. 4). 29 of the tested samples were positive (13%). *Plasmodium falciparum* was detected most frequently (n = 21, 72.4%), followed by *P. vivax* (n = 5, 17.2%) and *P. malariae* (n = 3, 10.3%) (Fig. 5). All malaria cases, for which information about the presumed country of infection was available (information available n = 17, 58.6%; no information available n = 12, 41.4%), had been acquired in Africa. Nigeria (n = 8, 47.1%) and Cameroon (n = 4, 23.5%) were most frequently reported as the country of infection. In eleven cases, information was provided on malaria chemoprophylaxis, which was not taken at all in ten cases and inappropriately in one case.

### *Anopheles* vectors in Erlangen—historical perspective

In 1922, Eckstein reported findings of *An. maculipennis* complex mosquitoes and *Anopheles bifurcatus* (currently known as *Anopheles claviger*) in moderate numbers in Erlangen [46].

### *Anopheles* vectors in Erlangen—present situation

**Fig. 6:**
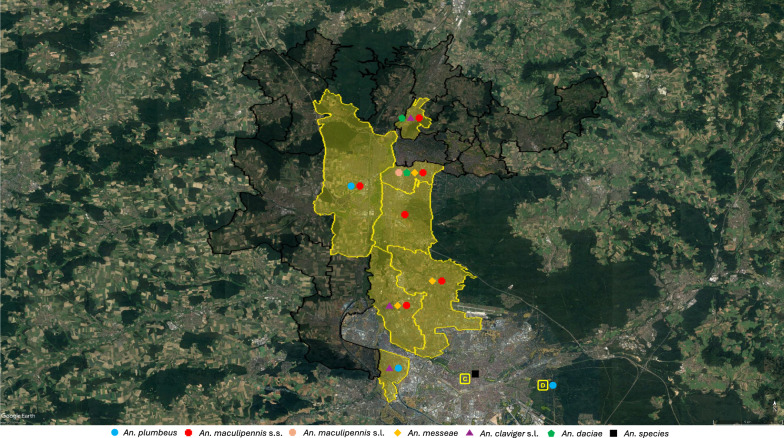
Schematic overview of areas with evidence for the presence of *Anopheles* species in/around Erlangen. Overview of the study area see Fig. 1. For a detailed list of the *Anopheles* species detected, see Table 4. Yellow, zip code area with detection of *Anopheles* species; black, zip code area without detection of *Anopheles* species; *An*., *Anopheles*; *s.s.*, sensu stricto; *s.l.*, sensu lato

In the course of two recent investigations, carried out by the Nürnberg Association of Entomologists (Nürnberger Entomologen), no *Anopheles* spp. were detected in biotopes within the city of Erlangen (2012 and 2013: “Brucker Lache” in the south, 2015: “Eisgrube” in the north) [47, 48]. By contrast, in the nearby metropolis of Nürnberg, *Anopheles plumbeus* was detected on the territory of the zoo in 1989 [49] and an *Anopheles* sample not further specified nearby the Imperial Castle in 2010/11 [50]. *Anopheles claviger *sensu lato (*s.l*.) and *An. plumbeus* were detected in the framework of mosquito surveillance in the district “Südstadt” of the nearby city of Fürth (zip code 90763) over the years 2021–2023 (Table 4). The query of the CULBASE database for the years 2011–2023 revealed the occurrence of *An. plumbeus*, *An. maculipennis *sensu stricto (*s.s*.), *Anopheles messeae*, *An. claviger s.l.* and *Anopheles daciae* within the city and in the surroundings of Erlangen (Table 4). The distribution of the species within the assessed zip code areas is shown in Fig. 6.

**Table 4:** *Anopheles* species detected in Erlangen and its surroundings, 2011–2023*

Postal code	Location	*Anopheles* species	Latitude	Longitude	Year
91088	Bubenreuth	*An. claviger s.l.*^a^	49.617790	11.005440	2016
91088	Bubenreuth	*An. daciae*^a^	49.624793	11.016777	2016
91088	Bubenreuth	*An. maculipennis s.s.*^a^	49.624793	11.016777	2016
91058	Erlangen	*An. maculipennis s.s.*^a^	49.571375	10.989186	2011
91058	Erlangen	*An. maculipennis s.s.*^a^	49.571375	10.989186	2017
91056	Erlangen	*An. maculipennis s.s.*^a^	49.554604	10.956356	2011
91056	Erlangen	*An. maculipennis s.s.*^a^	49.554604	10.956356	2013
91056	Erlangen	*An. maculipennis s.s.*^a^	49.570373	10.962922	2013
91056	Erlangen	*An. maculipennis s.s.*^a^	49.594525	10.966355	2012
91056	Erlangen	*An. plumbeus*^a^	49.600744	10.931640	2018
91052	Erlangen	*An. daciae*^a^	49.588160	10.997340	2012
91052	Erlangen	*An. maculipennis s.s.*^a^	49.588160	10.997340	2011
91052	Erlangen	*An. maculipennis s.s.*^a^	49.588160	10.997340	2011
91052	Erlangen	*An. maculipennis s.s.*^a^	49.588160	10.997340	2011
91052	Erlangen	*An. maculipennis s.s.*^a^	49.588160	10.997340	2011
91052	Erlangen	*An. maculipennis s.s.*^a^	49.588160	10.997340	2012
91052	Erlangen	*An. maculipennis s.s.*^a^	49.588160	10.997340	2013
91052	Erlangen	*An. maculipennis s.s.*^a^	49.588160	10.997340	2013
91052	Erlangen	*An. maculipennis s.s.*^a^	49.588160	10.997340	2014
91052	Erlangen	*An. maculipennis s.s.*^a^	49.588160	10.997340	2014
91052	Erlangen	*An. maculipennis s.l.*^a^	49.588160	10.997340	2015
91052	Erlangen	*An. messeae*^a^	49.588160	10.997340	2011
91052	Erlangen	*An. messeae*^a^	49.588160	10.997340	2012
91052	Erlangen	*An. messeae*^a^	49.588160	10.997340	2012
91052	Erlangen	*An. messeae*^a^	49.588160	10.997340	2013
91052	Erlangen	*An. messeae*^a^	49.588160	10.997340	2017
90765	Fürth	*An. claviger s.l.*^a^	49.495502	10.983886	2019
90765	Fürth	*An. claviger s.l.*^a^	49.495502	10.983886	2019
90765	Fürth	*An. maculipennis s.s.*^a^	49.486491	10.991604	2011
90765	Fürth	*An. maculipennis s.s.*^a^	49.486491	10.991604	2012
90765	Fürth	*An. maculipennis s.s.*^a^	49.486491	10.991604	2013
90765	Fürth	*An. maculipennis s.s.*^a^	49.486491	10.991604	2013
90765	Fürth	*An. maculipennis s.s.*^a^	49.486491	10.991604	2014
90765	Fürth	*An. maculipennis s.s.*^a^	49.486491	10.991604	2015
90765	Fürth	*An. messeae*^a^	49.486491	10.991604	2011
90765	Fürth	*An. messeae*^a^	49.486491	10.991604	2012
90765	Fürth	*An. messeae*^a^	49.486491	10.991604	2013
90765	Fürth	*An. messeae*^a^	49.486491	10.991604	2017
90763	Fürth	*An. claviger s.l.*^b^	49.452334	10.998046	2021
90763	Fürth	*An. claviger s.l.*^b^	49.452334	10.998046	2021
90763	Fürth	*An. claviger s.l.*^b^	49.458238	11.005101	2021
90763	Fürth	*An. plumbeus*^b^	49.45188	10.994084	2023
90763	Fürth	*An. plumbeus*^b^	49.452334	10.998046	2023
90763	Fürth	*An. plumbeus*^b^	49.452334	10.998046	2023
90763	Fürth	*An. plumbeus*^b^	49.457038	11.000358	2023
90763	Fürth	*An. plumbeus*^b^	49.455297	10.999826	2021
90763	Fürth	*An. plumbeus*^b^	49.458238	11.005101	2021
90480	Nürnberg	*An. plumbeus*^c^	n/d	n/d	1989 [49]
90427	Nürnberg	*An. maculipennis s.s.*^a^	49.531227	11.010851	2012
90427	Nürnberg	*An. maculipennis s.s.*^a^	49.531227	11.010851	2015
90427	Nürnberg	*An. messeae*^a^	49.531227	11.010851	2013
90403	Nürnberg	*An.* species^c^	n/d	n/d	2011 [50]

^*^Data sorted by postal code. ^a^German mosquito database at ZALF (CULBASE); ^b^Biogents AG; ^c^Kreis Nürnberger Entomologen e.V.; *An.*, *Anopheles*; *s.s.*, sensu strico; *s.l.*, sensu lato; n/d, no data

### Climate change—temperature in Erlangen

Complete temperature data for the Erlangen area is available for the years 1920–1930, 1947–1949 and 1951–2023 [38]. Accordingly, the following 20-year comparison periods can be defined: 1960–1979, 1980–1999 and 2000–2019. Between 1960–1979 and 2000–2019, the average monthly temperature rose by 1.6 °C (min. + 0.7 °C February, max. + 2.4 °C August; Table 5). An increase in the average annual number of months with an average temperature above 16 °C was also observed (1960–1979: 2.3, 2000–2019: 3.1). The number of years with three or more months with an average temperature above 16 °C per 20-year comparison period increased significantly (1960–1979: n = 9, 2000–2019: n = 19; p ≤ 0.001; 1960–1979 min. 1 month/year to max. 3 months/year, 2000–2019 min. 2 months/year to max. 4 months/year).

**Table 5:** Monthly average temperature in the Erlangen area per 20-year comparison period*

Monthly average temperature (°C)	1960–1979	1980–1999	2000–2019
January	−0.7	0.2	0.9
February	0.9	0.8	1.6
March	3.9	4.8	5.2
April	8.2	8.4	10.1
May	12.7	13.4	14.5
June	16.2	16.0	18.1
July	17.4	18.4	19.6
August	16.6	18.0	19.0
September	13.5	14.0	14.4
October	8.8	9.2	9.9
November	4.2	3.9	5.3
December	0.3	1.5	1.9

^*^The climatic data correspond to the monthly average air temperature (°C) at a height of 2 m (weather station 1279—Möhrendorf-Kleinseebach, dataset ID: urn:x-wmo:md:de.dwd.cdc::OBS_DEU_P1M_T2M) [38]

## Discussion

As in many parts of Germany, malaria used to occur endemically in Erlangen, a Bavarian city in the Nürnberg metropolitan area, until the end of the nineteenth century and afterwards disappeared. Recently, cases of autochthonous *P. vivax* infections were reported from southern European countries [23–26]. Therefore, the possibility of a re-emergence of autochthonous *Plasmodium* transmission is discussed for the city of Erlangen in Southern Germany, considering the local *Anopheles* populations, climate change and globalization.

### Perspectives on the change of malaria incidence in Erlangen

**Fig. 7:**
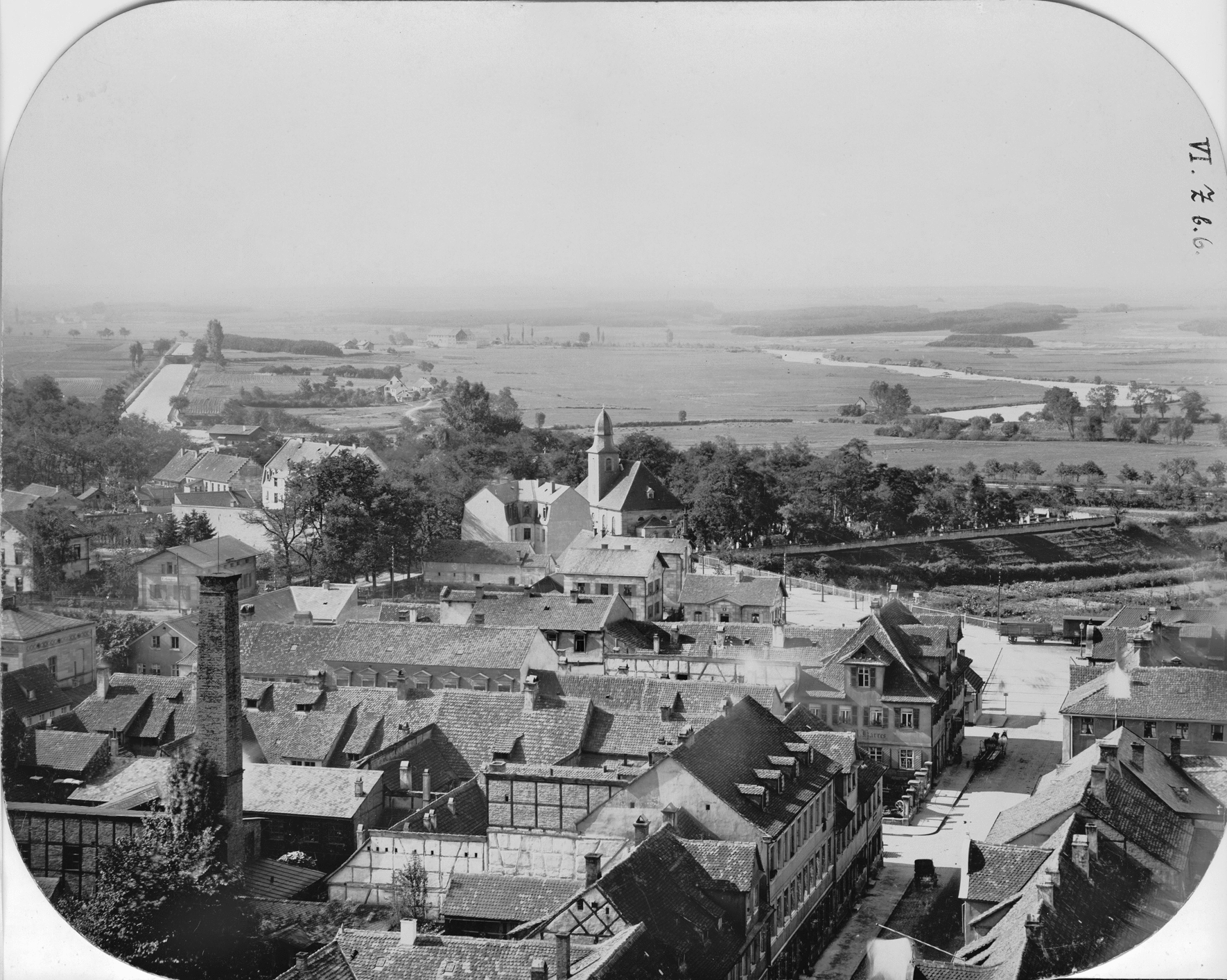
View from the Neustädter church-tower on the Southwestern suburb of Erlangen. Photographer Georg Daßler, 1891. Stadtarchiv Erlangen VI.Z.b.6

**Fig. 8:**
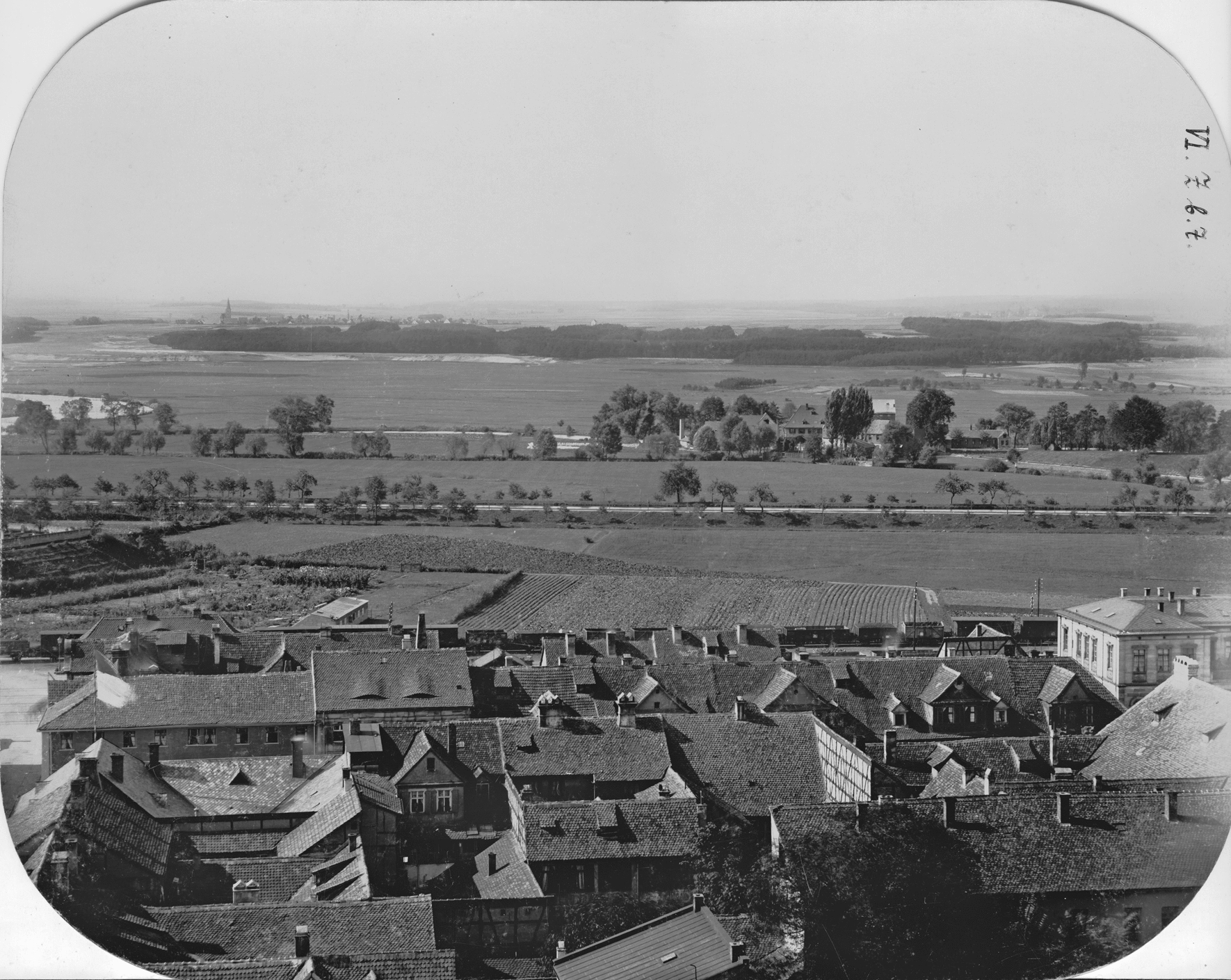
View from the Neustädter church-tower to the West. River Regnitz (left), the Ludwig-Danube-Main Canal (center) and, in the background, the Wöhrmühle. Photographer Georg Daßler, 1891. Stadtarchiv Erlangen VI.Z.b.7

In 1874, Dorsch, who worked as a public health officer in Erlangen, postulated that malaria had become established in Erlangen at the end of the 1820s. The increase of malaria cases in the Western parts of the city until the 1850s was considered partly due to the construction of the Ludwig-Danube-Main-Canal (connecting the rivers Main and Danube in the North and South of Bavaria, respectively) between 1836 and 1846 and the subsequent construction of the railroad line running between the canal and the city border. These construction measures hindered the out-flow of urban wastewater, and the surrounding meadows became marshy [51]. Compared to today's cityscape, it is noticeable that the Ludwig-Danube-Main-Canal at that time was located very close to the southwestern part of the city (Fig. 7), whereas nowadays a section of the highway A73 covers the area of the no longer existing canal. Between the railroad line and the canal, there was mainly farmland (Fig. 8).

**Fig. 9:**
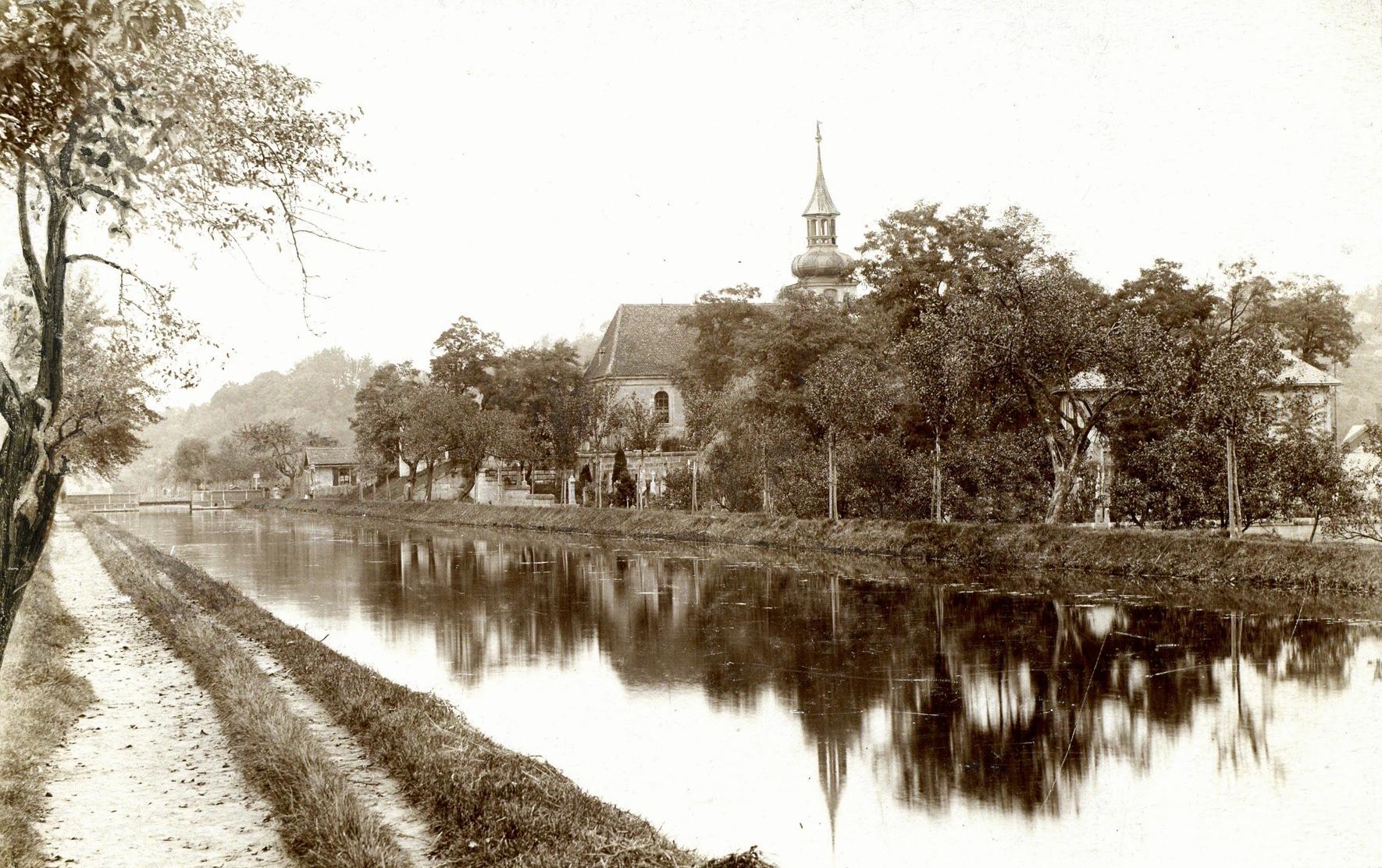
Ludwig-Danube-Main-Canal. View from South to North with the cemetery church at the so-called Martinsbühl. Photographer Leonhard Bergmann, ca. 1910. Stadtarchiv Erlangen VI.R.b.153

Extensive construction projects, especially close to waterways suitable for *Anopheles* development, have often been associated with short-term clusters of malaria [11]. A construction-related increase in personnel exchange with the regions of Upper Franconia in the North of Bavaria, which were considered malaria-rich due to intensive carp farming [11, 36, 44, 51], and increasing ground humidity of the surrounding area due to the clearing of forests [36] could have enhanced this development. It is not surprising that the low-lying districts of Erlangen, some of which are close to floodplains outside the city, had the highest relative numbers of cases (districts V and VI, Fig. 3) [36]. The canal, an artificial waterway with riparian vegetation and wide areas of stagnant water along its banks (Fig. 9), represented a suitable breeding site for the *An. maculipennis* complex, whose historical occurrence in Erlangen has been documented [46]. In addition to anti-malarials and insecticides, which caused a decrease in tertian malaria in Germany in general, some specific measures may have contributed to the disappearance of malaria in Erlangen at the end of the nineteenth century (Table 1). These are the reduction of breeding sites close to residential areas through, e.g., the regulation of inner-city plant nurseries in Erlangen in 1903 or the draining and restoration of groundwater wells and moats, as in the nearby town of Forchheim [44]. In neighbouring Nürnberg, a sudden rise of autochthonous tertian malaria cases occurred after World War II due to several exceptional circumstances, namely the return of numerous *Plasmodium*-infected soldiers [52], the destruction of sanitary and housing infrastructure, the creation of new mosquito breeding grounds [45] and the malnutrition and deterioration in medical care caused by the war [53]. A similar autochthonous transmission cycle has not been reported for post-war Erlangen, presumably, because the urban infrastructure was not significantly destroyed during World War II [54].

### *Anopheles* species in Germany and in the vicinity of the city of Erlangen

Mosquitoes of the *An. maculipennis* complex, especially *An. maculipennis s.s*., *Anopheles atroparvus* and *An. messeae*, account for the majority of historical *Plasmodium* transmissions and autochthonous malaria cases after World War II in Germany [29, 55]. *Anopheles daciae*, which was only recently separated from *An. messeae,* may have been responsible for (part of) *Plasmodium* transmissions attributed to *An. messeae,* although vector competence of *An. daciae* for *Plasmodium* transmission has not yet been demonstrated [56]. Species of the *An. maculipennis* complex native to Central Europe prefer to bite (farm) animals, but can also feed on humans [57], especially in cases of high mosquito population densities and when living areas and stables are in close proximity [46]. Since these *Anopheles* spp. still occur in Germany [29, 57–59] and are susceptible to African and Asian *P. vivax* strains [29], future autochthonous parasite transmission is possible, although the *Plasmodium* strains adapted to the local *Anopheles* populations by co-evolution have disappeared during the European malaria eradication campaigns of the first half of the twentieth century [12]. After a publication from 2016 suggested the occurrence of *An. messeae* and *An. daciae* within the greater Erlangen-Nürnberg area [56], the distribution of local *Anopheles* spp. was evaluated for the first time in the present study. Mainly taxa of the *An. maculipennis* complex (*An. maculipennis s.s.*, *An. messeae* and *An. daciae*) were detected by random citizen submissions to the “Mückenatlas” (Mosquito Atlas) project and during targeted field investigations, which confirms Eckstein's historical data [46]. In addition, the results of mosquito surveillance in the neighbouring city of Fürth revealed the occurrence of *An. plumbeus*, while *An. claviger s.l.* was found in the surroundings of Erlangen as well as in the city of Fürth, confirming the historical findings of *An. bifurcatus* [46] (Table 4, Fig. 6). Without further species differentiation, vector competence of *An. claviger s.l.* remains unclear as *Anopheles petragnani,* which was recently discovered to occur in Germany as well [60], is not able to transmit *Plasmodium* spp. as opposed to *An. claviger s.s.* [61]. Of the *Anopheles* spp. found, *An. maculipennis s.s*., *An. messeae* and *An. plumbeus* are considered potential vectors of *Plasmodium* spp. [57, 62].

### Malaria in the context of climate change and globalization

Since the development of both *Anopheles* and *Plasmodium* is temperature-dependent, climatic changes can influence malaria epidemiology. Models for the federal state of Lower Saxony [32], for the Southwest [30] or for all of Germany [63] have shown that the number of months with possible malaria parasite transmission could increase from 2 to up to 6 months per year in 2100 [32] as the temperature rises. The annual increase of months with an average temperature above 16 °C also applies to the Erlangen area. According to Becker [30], the observed rise in temperature could support the growth and spread of the *An. messeae* population in the city of Erlangen. Although climate change alone is unlikely to cause re-establishment of autochthonous malaria [31, 64], the climatic conditions may facilitate an increase in autochthonous malaria cases due to larger vector populations and faster sporozoite maturation [65].

**Fig. 10:**
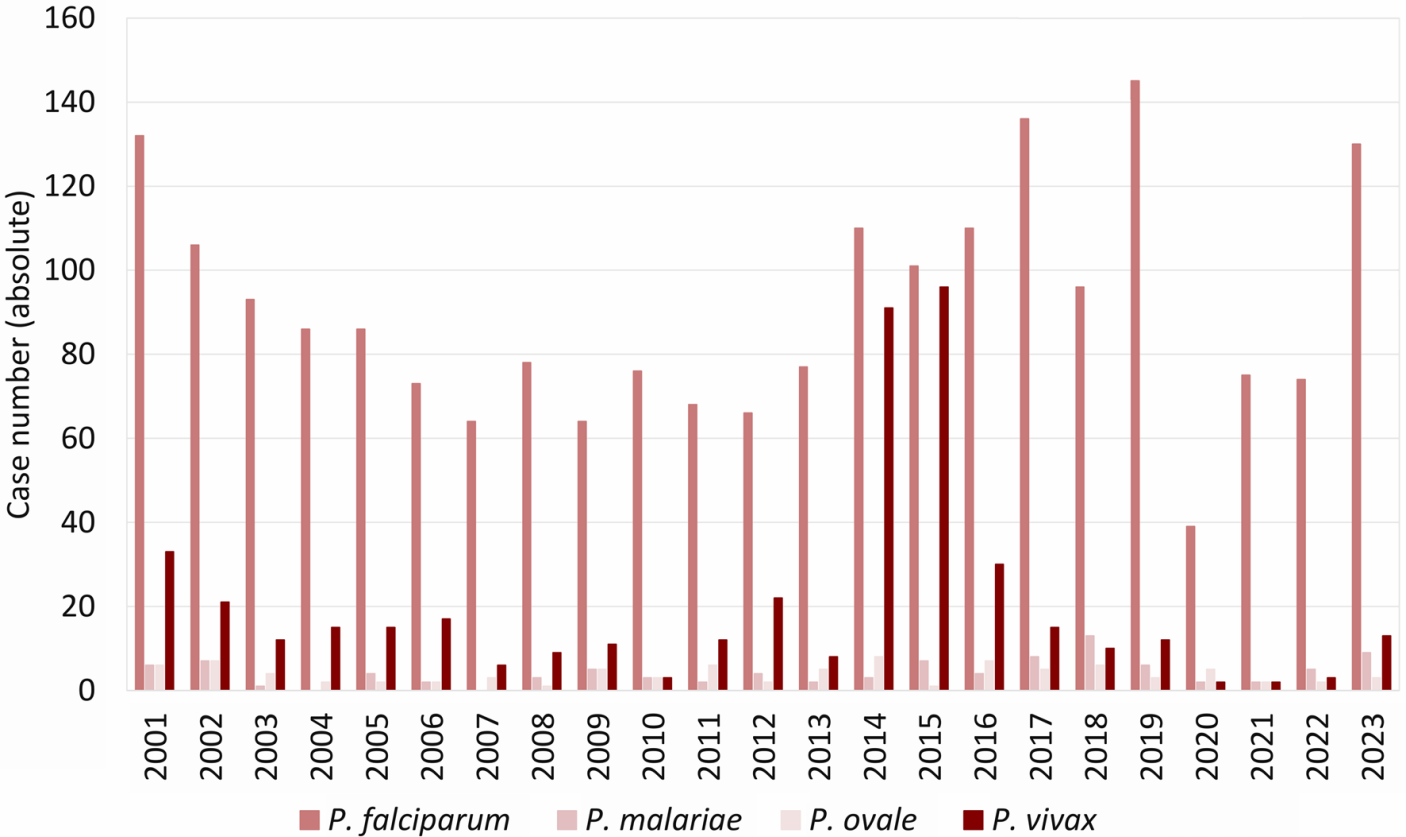
Cases of malaria in Bavaria, 2001–2023. *P.*, *Plasmodium.* Further data, e.g. detection of *Plasmodium* not identified to species level, are not shown due to the low number of cases. Robert Koch Institute: SurvStat@RKI 2.0, https://survstat.rki.de, data retrieved on March 18, 2024

Sequence analyses of autochthonously transmitted *P. vivax* strains in Spain showed that human immigration from malaria-endemic countries can lead to autochthonous transmission [66]. Immigration from Pakistan [67] and Eritrea [68, 69] led to a temporary increase in reported *P. vivax* infections in Germany. All of these were rated as imported and none as autochthonous infections. The temporary increase of *P. vivax*-infected immigrants was attributed to the exodus routes, traversing regions with risk of *Plasmodium* infection, whilst relapses of tertian malaria may be triggered by an increased incidence of bacterial infections due to deteriorating living conditions in the country of origin [69]. An increase of *P. vivax* infections was also noted in Bavaria (Fig. 10) and the University Hospital Erlangen in 2014 (Fig. 5). At least one of the *P. vivax* infections diagnosed in Erlangen was presumably acquired during the exodus from Eritrea to Germany via the Mediterranean Sea. As patients with relapsed tertian malaria can form a reservoir for autochthonous transmission, primaquine treatment of the hypnozoites is necessary, even if the infected person did not develop severe symptoms [70]. In the context of globalization, not only immigration may lead to the introduction of *Plasmodium* spp. to countries currently free of autochthonous malaria transmission, but also increased travelling activities, i.e. due to work, vacation or visiting family and friends. Introduction may occur due to infected travelers or due to passive transfer of infected *Anopheles* spp., i.e. as airport or baggage malaria [21, 22]. With respect to the malaria cases recently diagnosed in Erlangen, the anamnestic data (infection mostly acquired in West Africa, no or insufficient chemoprophylaxis) and the prevailing parasite species (mainly *P. falciparum*) are in line with the overall epidemiology in Germany [42]. The observed lack of sufficient chemoprophylaxis in *Plasmodium*-infected travelers returning from malaria-endemic areas may indicate low compliance or insufficient information on appropriate preventive measures, such as the use of insect repellents and bed nets against mosquito bites and the prescription and reliable intake of chemoprophylactic drugs. Therefore, a risk assessment prior to departure, i.e. by a general practitioner or an expert in travel medicine, is essential to prevent introduction of *Plasmodium* spp. by travellers [71].

### Limitations

The present work has some limitations. When analysing medical-historical sources, the retrospective diagnoses such as malaria must be viewed critically [72]. As no clinical specimens are available, the historical descriptions can only be checked for their plausibility. At least up to the year 1880, Mayr [36] only used data based on clinical diagnoses, as neither the etiology of malaria was known nor had *Anopheles* mosquitoes been identified as the vectors of the parasite at that time [73]. Typhoid diseases in particular could have been mistaken for malaria if the course of fever was atypical. Such an error was, for example, reported in the North Rhine-Westphalian town of Arnsberg in 1903 [44]. Therefore, the possibility that other diseases accounted for some of the malaria cases in Erlangen cannot be ruled out. However, since malaria used to be a relatively common disease, a good clinical diagnostic quality can be assumed. Therapeutic success through the administration of quinine may also have helped the treating physicians in diagnosing malaria *ex juvantibus*. Mayr explicitly focused on in-patients, as the quality of diagnosis was assumed to be most reliable with those [36]. The monthly infectious disease data from the “Erlanger Tagblatt” can supplement this data with reports from the out-patient sector, i.e. the entire district of Erlangen. The exemplary evaluation of the year 1899 shows that the number of malaria cases reported there (in relation to the total number of reported illnesses) was comparable to the number of cases treated in hospital in the 1880s (Table 1). In nineteenth century, malaria was not a notifiable pathogen in Germany and, unfortunately, there are no case reports available from the local health authorities (Erlangen City Archive 9.A.1241) [74]. Finally, as shown in Fig. 6, *Anopheles* spp. were especially detected in urban areas. This might reflect a selection bias due to higher frequencies of mosquito submissions to the “Mückenatlas” (Mosquito Atlas) by submitters with an urban residence or due to the locations chosen for targeted field studies. It is likely that *Anopheles* spp. are also present in rural municipalities around the city of Erlangen, possibly reaching the city area [40].

## Conclusion

Malaria occurred endemically in Erlangen in the past and represented—probably due to major construction projects at the beginning of the nineteenth century—a considerable of in-patient diagnoses around 1850, but disappeared at the beginning of the twentieth century. The annual number of months with an average temperature above 16 °C, which enables the extrinsic development of *P. vivax*, has risen continuously in the Erlangen area in recent decades. With *An. messeae*, *An. maculipennis s.s.* and *An. plumbeus,* three potential vectors of tertian malaria parasites have been detected, whereas the role of *An. daciae* in *Plasmodium* transmission still remains unclear. Findings of *An. claviger s.l.* require further differentiation on species level to make a statement about vector competence. Autochthonous tertian malaria cases in neighbouring Nürnberg after World War II show that the re-emergence of malaria around Erlangen is principally possible under extreme conditions. However, as long as the excellent sanitary and medical conditions are maintained and the number of malaria patients remains low, a re-establishment of autochthonous malaria in Erlangen is unlikely. The situation in Erlangen is comparable to the assumptions for Germany in general [12]. In order to avoid an unexpected increase of malaria cases, surveillance measures are necessary [75] and environmental changes, e.g., renaturation measures, need to take into account unwanted effects on the expansion of mosquito populations [76]. General practitioners must provide sufficient medical information for patients who plan to travel to countries with known risks of *Plasmodium* infection and always need to consider malaria in patients with fever after return from endemic areas [77]. The Bavarian mosquito surveillance carried out by the federal authorities, the “Mückenatlas” (Mosquito Atlas) as a nationwide citizen science project and private-sector measures offer various possibilities for multi-layered mosquito monitoring. Based on the potential *Plasmodium* vectors found so far, a standardized assessment of the mosquito species occurring in the metropolitan area of Erlangen-Nürnberg could be useful in order to obtain baseline data for the identification of future changes of *Anopheles* populations and for an improved assessment of the risk of (re-) emerging infectious diseases like tertian malaria.

## Data Availability

No datasets were generated or analysed during the current study.

## References

[1] WHO. World malaria report 2023. Geneva: World Health Organization; 2023.

[2] Mantilla-Flórez YF, Barragán BP, Tuta-Quintero EA, Pérez-Díaz CE. *Plasmodium vivax* infection due to percutaneous exposure in non-endemic area. Infect Dis Health. 2020;25:60–2. 31481333 10.1016/j.idh.2019.08.001

[3] Rosso F, Agudelo Rojas OL, Suarez Gil CC, et al. Transmission of malaria from donors to solid organ transplant recipients: a case report and literature review. Transpl Infect Dis. 2021;23:e13660. 34057797 10.1111/tid.13660

[4] Börsch G, Odendahl J, Sabin G, Ricken D. Malaria transmission from patient to nurse. Lancet. 1982;2:1212. 6128508 10.1016/s0140-6736(82)91219-3

[5] Gonzalez Garcia JJ, Arnalich F, Peña JM, et al. An outbreak of *Plasmodium vivax* malaria among heroin users in Spain. Trans R Soc Trop Med Hyg. 1986;80:549–52. 3544357 10.1016/0035-9203(86)90136-7

[6] Pritt BS. Plasmodium and Babesia. In: Carrol KC, Pfaller MA, editors. Manual of clinical microbiology. Washington DC: American Society for Microbiology Press; 2023. p. 2757–76.

[7] Boualam MA, Pradines B, Drancourt M, Barbieri R. Malaria in Europe: a historical perspective. Front Med. 2021;8:691095. 10.3389/fmed.2021.691095 PMC8277918 34277665

[8] Wernsdorfer WH. [Malaria in Central Europe] (in German). Denisia. 2002;6:201–12.

[9] Evangelia-Theophano P. Malaria eradication in the European World: historical perspective and imminent threats. In: Manguin S, Dev V, editors. Towards malaria elimination - a leap forward. IntechOpen: Rijeka; 2018.

[10] Maier WA. [The disappearance of swamp fever in Europe: coincidence or necessity?] (in German). Denisia. 2004;13:515–27.

[11] Trautmann A. [The spread of indigenous malaria in Germany in the past and present. (A compilation based on literature reports and official medical data)] (in German). Arch Hyg. 1913;80:84–108.

[12] Kampen H. [Will malaria become a threat to Europe again?] (in German). In: Lozán JL, Grassl H, Karbe L, Jendritzky G (eds). Warnsignal Klima: Gefahren für Pflanzen, Tiere und Menschen Elektron. 2014 . https://www.klima-warnsignale.uni-hamburg.de/wp-content/uploads/pdf/de/gesundheitsrisiken/warnsignal_klima-gesundheitsrisiken-kapitel-3_2_2.pdf. Accessed 14 Aug 2024.

[13] Wollgramm DB. [The spreading and control of autochthonous malaria in Germany- 1850 to 1900] (in German). Düsseldorf: Heinrich-Heine-Universität Düsseldorf, Dissertation. 2016. https://docserv.uni-duesseldorf.de/servlets/DocumentServlet?id=40578. Accessed 13 Aug 2024.

[14] Kampen H, Kronefeld M, Werner D. Culicid mosquitoes as vectors of disease agents in Europe. In: Mehlhorn H, editor. Arthropods as vectors of emerging diseases. 3rd ed. Berlin: Spinger; 2012. p. 1–30.

[15] Kemper SA. [Autochthonous malaria in Germany from 1900 - 1951 its spread and disappearance] (in German). Düsseldorf: Heinrich-Heine-Universität Düsseldorf, Dissertation. 2021. https://docserv.uni-duesseldorf.de/servlets/DocumentServlet?id=56259. Accessed 13 Aug 2024.

[16] Güllenstern M-L. [Malaria in Germany at the time of the Second World War and the first post-war years] (in German). Würzburg: Julius-Maximilians-Universität Würzburg, Dissertation; 1990.

[17] Conroy MS. Malaria in late tsarist Russia. Bull Hist Med. 1982;56(1):41–55. 7046859

[18] Huldén L, Huldén L, Heliövaara K. Endemic malaria: an “indoor” disease in northern Europe. Hist Data Anal Malar J. 2005;4(1):19. 10.1186/1475-2875-4-19 PMC1090613 15847704

[19] WHO. Countries and territories certified malaria-free by WHO. Geneva, World Health Organization, 2024. https://www.who.int/teams/global-malaria-programme/elimination/countries-and-territories-certified-malaria-free-by-who. Accessed 15 Nov 2024.

[20] Tatem AJ, Jia P, Ordanovich D, Falkner M, Huang Z, Howes R, et al. The geography of imported malaria to non-endemic countries: a meta-analysis of nationally reported statistics. Lancet Infect Dis. 2017;17:98–107. 27777030 10.1016/S1473-3099(16)30326-7 PMC5392593

[21] Isaäcson M. Airport malaria: a review. Bull World Health Organ. 1989;67:737–43. 2699278 PMC2491318

[22] Alenou LD, Etang J. Airport malaria in non-endemic areas: new insights into mosquito vectors, case management and major challenges. Microorganisms. 2021;9:2160. 34683481 10.3390/microorganisms9102160 PMC8540862

[23] Romi R, Boccolini D, Menegon M, Rezza G. Probable autochthonous introduced malaria cases in Italy in 2009–2011 and the risk of local vector-borne transmission. Euro Surveill. 2012;17:20325. 23218391

[24] Santa-Olalla Peralta P, Vazquez-Torres MC, Latorre-Fandos E, Mairal-Claver P, Cortina-Solano P, Puy-Azón A, et al. First autochthonous malaria case due to *Plasmodium vivax* since eradication, Spain, October 2010. Euro Surveill. 2010;15:19684. 20961517 10.2807/ese.15.41.19684-en

[25] Andriopoulos P, Economopoulou A, Spanakos G, Assimakopoulos G. A local outbreak of autochthonous *Plasmodium vivax* malaria in Laconia, Greece–a re-emerging infection in the southern borders of Europe? Int J Infect Dis. 2013;17:e125–8. 23098813 10.1016/j.ijid.2012.09.009

[26] Spanakos G, Alifrangis M, Schousboe ML, Patsoula E, Tegos N, Hansson HH, et al. Genotyping *Plasmodium vivax* isolates from the 2011 outbreak in Greece. Malar J. 2013;12:463. 24373457 10.1186/1475-2875-12-463 PMC3877964

[27] Centers for Disease Control and Prevention. Locally acquired malaria cases identified in the United States. 2023. https://emergency.cdc.gov/han/2023/han00494.asp#print. Accessed 15 Nov 2024.

[28] Robert LL, Santos-Ciminera PD, Andre RG, Schultz GW, Lawyer PG, Nigro J, et al. *Plasmodium*-infected *Anopheles* mosquitoes collected in Virginia and Maryland following local transmission of *Plasmodium vivax* malaria in Loudoun County, Virginia. J Am Mosq Control Assoc. 2005;21:187–93. 16033121 10.2987/8756-971X(2005)21[187:PAMCIV]2.0.CO;2

[29] Jetten TH, Takken W. Anophelism without malaria in Europe: a review of the ecology and distribution of the genus *Anopheles* in Europe. Wageningen Agric Univ Pap. 1994;94:5.

[30] Becker N. [The role of globalization and climate change on the development of mosquitoes and their transmitted diseases in Central Europe] (in German). Environ Sci Eur. 2009;21:212–22.

[31] Krimkowski J. [The advance of malaria into Central Europe in the wake of global warming: Germany as a case study] (in German). Stuttgart: ibidem-Verl; 2011.

[32] Schröder W, Schmidt G. Modelling potential malaria spread in Germany by use of climate change projections. A risk assessment approach coupling epidemiologic and geostatistical measures. Cham: Springer; 2014.

[33] Bavarian State Office for Statistics. Update of the population status (Table 12411–003r). 2025. https://www.statistikdaten.bayern.de/genesis/online?operation=result&code=12411-003r. Accessed 02 Jan 2025.

[34] Bavarian State Office for Statistics. Municipal statistics 2022. District-free city of Erlangen 09 562. A selection of important statistical data, 2023.

[35] [Bavarian local news - Erlangen] (in German). Erlanger Tagblatt. 1899.

[36] Mayr E. [The Malaria in Erlangen over the past 30 years] (in German). Nürnberg: Friedrich-Alexander-Universität Erlangen, Dissertation; 1889.

[37] Stöcklein H. Map of Erlangen. Erlangen; Nürnberg: Blaesing; Schmidtner; 1890.

[38] DWD Climate Data Center (CDC). Monthly average of station measurements of air temperature at a height of 2 m in °C for Germany. https://cdc.dwd.de/portal/. Accessed 17 Oct 2023.

[39] Walther D, Kampen H. The Citizen Science Project ‘Mueckenatlas’ helps monitor the distribution and spread of invasive mosquito species in Germany. J Med Entomol. 2017;4:1790–4. 10.1093/jme/tjx166 PMC5850493 29029273

[40] Verdonschot PFM, Besse-Lototskaya AA. Flight distance of mosquitoes (Culicidae): a metadata analysis to support the management of barrier zones around rewetted and newly constructed wetlands. Limnologica. 2014;45:69–79.

[41] Salahimoghadam A, Khoshdel A, Barati M, Sedaghat MM. An overview and mapping of malaria and its vectors in Iran. Hormozgan Med J. 2014;18:428–40.

[42] Falkenhorst GE, Enkelmann J, Faber M, Brinkwirth S, Lachmann R, Bös L, et al. [The situation of important infectious diseases - Imported Infectious diseases 2022](in German). Epidemiol Bull. 2023;46:3–20.

[43] Penzoldt F. [The Malaria in Erlangen] (in German). Sitzungsberichte der Physikalisch-Medizinischen Sozietät zu Erlangen. 1883;16:67–75.

[44] Schuberg A. [The present and past occurrence of malaria and the distribution of anopheles mosquitoes in the territory of the German Reich] (in German). Arbeiten aus dem Reichsgesundheitsamte; 1927.

[45] Steib H. [Endemic malaria tertiana in Nuremberg] (in German). Erlangen: Friedrich-Alexander-Universität Erlangen, Dissertation; 1949.

[46] Eckstein F. [The distribution of *Anopheles* in Bavaria and its suspected importance for the introduction of malaria](in German). J Appl Entomol. 1922;8:229–82.

[47] von der Dunk K. [Studies on entomological diversity in the Brucker Lache, a special habitat that has been protected for over 50 years in the immediate vicinity of the city of Erlangen] (in German). Galathea, Berichte des Kreises Nürnberger Entomologen eV. 2014;30:5–48.

[48] von der Dunk K, Brünner K. [Entomological investigations in the habitat “Eisgrube”, a forest section on the northern slope of the Burgberg near Erlangen (Middle Franconia, Northern Bavaria)] (in German). Galathea, Berichte des Kreises Nürnberger Entomologen eV. 2015;31:5–22.

[49] von der Dunk K, Kraus M. [Fundamental studies on the diverse insect fauna of the Tiergarten Nuremberg with special emphasis on the Hymenoptera] (in German). Beiträge zur Bayerischen Entomofaunistik. 2014;13:67–207.

[50] von der Dunk K, Köstler W, Tannert R, Weltner L. [Recording the insect fauna of Nuremberg’s Imperial Castle for the “Castle Habitat” project as part of the implementation of the Bavarian Biodiversity Strategy] (in German). Galathea, Berichte des Kreises Nürnberger Entomologen eV. 2011;27:93–140.

[51] Dorsch G. [Causes of malaria in Erlangen and the surrounding area] (in German). Deutsche Klinik. 1874;11:83–5.

[52] Merkel H. [About native endogenous malaria in Germany] (in German). Erlangen: Friedrich-Alexander-Universität Erlangen, Dissertation; 1951.

[53] Weinreb A. “For the hungry have no past nor do they belong to a political party”: debates over German Hunger after World War II. Cent Eur Hist. 2012;45:50–78.

[54] Lehmann G. Second World War (in German). In: Friederich C, von Haller B, Jakob A, editors. Erlanger Stadtlexikon. Tümmels: Nürnberg; 2002.

[55] Military Government of Germany - Public Health and Medical Affairs. Monthly Report of the Military Governor, US Zone. 20 July 1946 No. 12. 1946.

[56] Kampen H, Schäfer M, Zielke DE, Walther D. The *Anopheles maculipennis* complex (Diptera: Culicidae) in Germany: an update following recent monitoring activities. Parasitol Res. 2016;115:3281–94. 27444437 10.1007/s00436-016-5189-9

[57] Bertola M, Mazzucato M, Pombi M, Montarsi F. Updated occurrence and bionomics of potential malaria vectors in Europe: a systematic review (2000–2021). Parasit Vectors. 2022;15:88. 35292106 10.1186/s13071-022-05204-y PMC8922938

[58] Kampen H, Werner D. [The recurring necessity of mosquito surveillance and research] (in German). Bundesgesundheitsbl. 2015;58:1101–9. 10.1007/s00103-015-2218-2 26335745

[59] Werner D, Kowalczyk S, Kampen H. Nine years of mosquito monitoring in Germany, 2011–2019, with an updated inventory of German culicid species. Parasitol Res. 2020;119:2765–74. 32671542 10.1007/s00436-020-06775-4 PMC7431392

[60] Becker N, Pfitzner WP, Czajka C, Kaiser A, Weitzel T. *Anopheles* (*Anopheles*) *petragnani* Del Vecchio 1939-a new mosquito species for Germany. Parasitol Res. 2016;115:2671–7. 27003404 10.1007/s00436-016-5014-5 PMC4914522

[61] Martínez-Barciela Y, Polina A, Garrido J. New contributions to the knowledge of two riparian mosquitoes in northwestern Spain: *Anopheles petragnani* and *Culex mimeticus* (Diptera: Culicidae). Environ Entomol. 2024;53:619–28. 38909379 10.1093/ee/nvae061 PMC11329618

[62] Kampen H, Walther D. Vector potential of mosquito species (Diptera: Culicidae) occurring in Central Europe. In: Benelli G, Mehlhorn H, editors. Mosquito-borne diseases: implications for public health. Cham: Springer International Publishing; 2018. p. 41–68.

[63] Holy M, Schmidt G, Schröder W. Potential malaria outbreak in Germany due to climate warming: risk modelling based on temperature measurements and regional climate models. Environ Sci Pollut Res Int. 2011;18:428–35. 20809105 10.1007/s11356-010-0388-x

[64] Lindsay SW, Hole DG, Hutchinson RA, Richards SA, Willis SG. Assessing the future threat from vivax malaria in the United Kingdom using two markedly different modelling approaches. Malar J. 2010;9:70. 20205713 10.1186/1475-2875-9-70 PMC2845590

[65] Fischer L, Gültekin N, Kaelin MB, Fehr J, Schlagenhauf P. Rising temperature and its impact on receptivity to malaria transmission in Europe: a systematic review. Travel Med Infect Dis. 2020;36:101815. 32629138 10.1016/j.tmaid.2020.101815

[66] Barrado L, Ezpeleta C, Rubio JM, Martin C, Azcona JM, Arteaga M, et al. Source identification of autochthonous-introduced *Plasmodium vivax* malaria, Spain. Infection. 2017;45:111–4. 27565658 10.1007/s15010-016-0941-8

[67] Stark K, Schöneberg I. Increase in malaria cases imported from Pakistan to Germany in 2012. Euro Surveill. 2012;17:20320. 23231855 10.2807/ese.17.47.20320-en

[68] Vygen-Bonnet S, Stark K. Changes in malaria epidemiology in Germany, 2001–2016: a time series analysis. Malar J. 2018;17:8. 29334944 10.1186/s12936-018-2175-y PMC5769339

[69] Sondén K, Rolling T, Wångdahl A, Ydring E, Vygen-Bonnet S, Kobbe R, et al. Malaria in Eritrean migrants newly arrived in seven European countries, 2011 to 2016. Euro Surveill. 2019;24:1800139. 30722809 10.2807/1560-7917.ES.2019.24.5.1800139 PMC6386211

[70] Roggelin L, Tappe D, Noack B, Addo MM, Tannich E, Rothe C. Sharp increase of imported *Plasmodium vivax* malaria seen in migrants from Eritrea in Hamburg, Germany. Malar J. 2016;15:325. 27316351 10.1186/s12936-016-1366-7 PMC4912711

[71] Agudelo Higuita NI, White BP, Franco-Paredes C, McGhee MA. An update on prevention of malaria in travelers. Ther Adv Infect Dis. 2021;8:20499361211040690. 34484736 10.1177/20499361211040690 PMC8408895

[72] Leven K-H. Malaria. In: Leven K-H, editor. Ancient medicine: an encyclopedia. München: C. H. Beck; 2005.

[73] Cox FEG. History of the discovery of the malaria parasites and their vectors. Parasit Vectors. 2010;3:5. 20205846 10.1186/1756-3305-3-5 PMC2825508

[74] Königliches Staatsministerium des Innern (ed.). [Notification of infectious diseases to the royal military authorities] (in German). München; 1889.

[75] Dalitz MK. [Autochthonous malaria in central Germany] (in German). Halle: Martin-Luther-Universität Halle-Wittenberg, Dissertation; 2005. 10.25673/2444. Accessed 13 Aug 2024.

[76] Bavarian Environment Agency. [Mosquitoes in renaturalized moors in Bavaria 2020. Investigation of mosquitoes on selected renaturalized and degraded moorland areas in raised bogs and fens in comparison to adjacent settlement areas] (in German). München; 2021.

[77] Calderaro A, Montecchini S, Buttrini M, Piccolo G, Rossi S, Arcangeletti MC, et al. Malaria diagnosis in non-endemic settings: the European experience in the last 22 years. Microorganisms. 2021;9:2265. 34835391 10.3390/microorganisms9112265 PMC8620059

[78] Hormann H. [Malaria in Germany 1945–1947] (in German). Z Tropenmed Parasitol. 1949;1:31–91.

[79] Heber U. [Malaria in Franconia during the Second World War and the first post-war years](in German). Würzburg: Julius-Maximilians-Universität Würzburg, Dissertation; 1990.

